# Microbial allies: shaping growth, physiology, and rhizosphere dynamics of onion (*Allium cepa* L.)

**DOI:** 10.7717/peerj.20566

**Published:** 2026-01-06

**Authors:** Pranjali A. Gedam, Kiran Khandagale, Vitthal T. Barvkar, Snehal Bhandari, Sucheta Patil, Sagar Wayal, Indira Bhangare, Kiran P. Bhagat, Kiran Landage, Rajiv Kale, Vivek Bhoite, Sanket More, Vijay Mahajan, Suresh Gawande

**Affiliations:** 1 ICAR-Directorate of Onion and Garlic Research, Pune, India; 2 Department of Botany, Savitribai Phule Pune University, Pune, India; 3 HPC-Medical and Bioinformatics Applications Group, Centre for Development of Advanced Computing, Pune, India; 4 ICAR-Directorate of Floriculture Research, Pune, India; 5 Plant Pathology Department, College of Agriculture, Pune, India; 6 Soil Science Department, Krishi Vidnyan Kendra, Baramati, India

**Keywords:** Onion, Phenotypic growth, Physiology, PGPM, Metagenomics, Soil microbial diversity

## Abstract

The present study investigates the dual impact of microbial biofertilizers on the phenotypic performance and rhizosphere microbiome composition in an onion crop. A pot experiment was conducted with seven treatments of microbial inoculants, such as Azotobacter, Azospirillum, *Piriformospora indica*, phosphate solubilizing bacteria (PSB), and control treatments with and without chemical fertilizers. The growth, physiological, and biochemical traits of onion were assessed alongside rhizospheric soil microbiome profiling using 16S rRNA metagenomic sequencing. Significant enhancement in plant height, leaf number, leaf area, chlorophyll content, photosynthetic rate, and antioxidant enzyme activity with low leaf temperature was observed in plants inoculated with Azotobacter and Azospirillum. Notably, the Azotobacter treatment yielded a significant enhancement in the bulb phenol content. Rhizosphere metagenomic analysis revealed 17 dominant phyla, with *Actinobacteria* (25.3%), *Proteobacteria* (22.2%), *Firmicutes* (12.8%), and *Chloroflexi* (11.02%) comprising over 70% of the total microbiome. Alpha and beta diversity metrics indicated that microbial inoculation, especially with Azospirillum and PSB, enriched the soil microbial community structure. Distinct clustering and correlations with specific microbial taxa such as *Candidatus Nitrososphaera* and *Pseudomonas* were observed in response to individual biofertilizer treatments. This study highlights the potential of biofertilizers not only in enhancing onion growth and development but also in modulating beneficial rhizosphere microbial communities. Integrating biofertilizers into onion production systems could reduce the dependency on chemical fertilizers and promote sustainable crop management.

## Introduction

Bulb onion is an economically important vegetable crop cultivated globally, contributing nearly 24% of the world’s vegetable production (Gasser et al., 2023). India ranks first in the area and production of bulb onions with an average production of 31.68 million tons from a 1.94 million hectare area (FAO, 2024). Onion production seems to be increased by 5–6 folds and area by 3-fold from 2001 to 2022 (Kale et al., 2024). In spite of the substantial increase in area and production, the overall onion productivity is low at 16.32 t/ha^−1^ in India compared to world average that is 18.53 t/ha^−1^ (Arunachalam et al., 2024). Compared to other vegetable crops, onion gains chief importance due to its high economic, nutritional, culinary, and medicinal values (Khandagale & Gawande, 2019). In India, it thus becomes a national strategic vegetable crop due to its large consumption and huge demand in the national and international market.

To meet the continuously growing demand, farmers make use large quantity of inorganic fertilizers to increase the onion bulb production. Traditionally, inorganic fertilizers are the major source of soil nutrients that are extensively used in crop production. However, the overuse of these chemical fertilizers has led to an imbalance in nutrient supply and its utilization. It not only affects the cultivatable area but also pollutes the ecosystem. Additionally, it deteriorates the soil quality by adversely affecting the nutrient cycle and inhabitant’s beneficial microbes eventually increasing toxicity to plant and decreasing soil fertility (Tayoh, Kiyo & Nkemnyi, 2016). Thus, the foremost concern in onion production is to improve soil health and simultaneously achieve the potential bulb production.

The fertilizer recommendations by the ICAR-Directorate of Onion and Garlic Research (ICAR-DOGR) to achieve a potential onion bulb yield of 40–50 tons per hectare in the *Rabi* season is 110:40:60:30 kg NPKS and 15 tons FYM per hectare (Thangasamy & Lawande, 2015). This is a sign of the huge demand for nutrients by the onion crop to achieve the potential yield. However, being a shallow-rooted crop, it faces challenges in nutrient uptake that varies with the crop growth stage (Brewster, 2008). Most of the nutrients, particularly nitrogen, leach beyond the root zones or denitrification of excess nitrogen takes place, making it unavailable to the plant. The only way out to minimize this deleterious effect of chemical fertilizers and maximize the nutrient use efficiency is by applying the nutrients directly near the root zone or make use of plant growth promoting microbes (PGPM) or biofertilizers. PGPM interact with the plant and improve its growth, development and nutrient uptake (Pii et al., 2015). Most of the essential nutrients for plants, such as nitrogen, phosphorus, potassium, zinc and silica, are present in complex insoluble forms in the soil. Microbes help in solubilizing these nutrients and making them easily available to plants (Nosheen, Ajmal & Song, 2021). Thus, it can effectively reduce the use of inorganic fertilizers and improve the soil health to achieve healthy plant growth. It enriches the soil by increasing the microbial diversity that interact with plants and build a dynamic community may be beneficial endophytic bacteria, native microbes or disease-causing microbial agents (Schirawski & Perlin, 2018). It can invade all plant tissues, including the rhizosphere where the microbial population flourishes and promotes plant growth by improving the supply and availability of nutrients or by inducing defence response against several pest and diseases (Elnahal et al., 2022). Additionally, they solubilize the fixed nutrients in the soil and make them easily available to plants. They help in root growth and development, which ultimately improves the water and nutrient uptake capacity of plants (Singh et al., 2020). They can be directly applied to the soil or through root inoculation and seed treatment where they can multiply rapidly to develop a large population in the rhizosphere. Nowadays, advancing applied research through the use of PGPM is of special interest for improving crop productivity and soil health. The major PGPMs used in crop production are Azotobacter, Azospirillum, Trichoderma, Vesicular Arbuscular Mycorrhizae, Blue green algae, Potassium Mobilizing bacteria (KMB) and Phosphorus Solubilizing microbes (PSB) (Malgioglio et al., 2022). Recently, *Piriformospora indica* (*P. indica*), an endophytic root-colonizing fungus species, which belongs to Sebacinales (Basidiomycota) has gained significant attention owing to its unique characteristics of augmenting nutrient acquisition, stimulating plant growth, and enhancing plant resilience against biotic and abiotic stresses (Roylawar et al., 2021; Li et al., 2023a; Yalamalle et al., 2024). Every crop responds differently to a particular type of microbe or group of microbes. Researchers are working constantly to understand the microbial diversity and their interaction with the soil and environment. However, there are several microbial populations that can’t be cultured in the research laboratory. The only way to study the microbial diversity in soil is by using the recently developed next-generation DNA sequencing (NGS) technology, such as the metagenomics analysis. This technology created an opportunity to advance our knowledge of the function of soil microbial communities, diversity and plant-microbe interaction in a particular environment (Khandagale et al., 2020; Oliveira et al., 2022). In this method, the total DNA present in particular environmental sample was analysed to understand the microbial and environmental diversity. This will help to identify the unique physiological and metabolic processes linked with the microbial communities and the positive correlation that exists between the soil quality and the presence of particular groups of microbial populations. Several studies have demonstrated the significant role played by PGPM in improving crop growth by establishing a powerful plant-microbial association. However, a limited number of studies have been reported on onion crop in relation to plant microbial association and their beneficial effect in relation to bulb yield and quality (Tinna et al., 2020; Pellegrini et al., 2021; Younes et al., 2023; Arunachalam et al., 2024).

To address this issue, the present study was designed with a hypothesis that the application of particular microbial inoculants may enhance onion crop growth and soil health compared to chemical fertilizers. Secondly microbial inoculation may alter the microbial diversity in the rhizosphere of the onion crop. To validate the hypothesis, the study was designed with an objective to evaluate the effect of different microbial inoculants on onion crop growth and soil microbial diversity. The study examined the impact of the certain microbial inoculants, namely, Azotobacter, Azospirillum, KMB, PSB, and *P. indica*, on the microbial community structure of the onion rhizosphere. The metagenomics approach implemented in this study is the most reliable method for examining the microbial diversity in the onion rhizosphere in response to microbial treatments. The results from the present work will further help to identify the promising microbes that can be utilized to minimize the use of chemical fertilizers in the onion cropping system. Furthermore, the present work highlighted the microbial association with the increase in onion crop growth, bulb yield, quality, and soil health.

## Materials and Methods

### Bio-inoculants

Pure microbial cultures of *Azospirillum brasilense* (AZOS), *Azotobacter chroococcum* (AZOT), and phosphorus solubilizing bacteria (*Bacillus megaterium*) (PSB) were obtained from the College of Agriculture, Pune, Maharashtra, India. A pure culture of *Piriformospora indica* (PI) was obtained from Dr. J. Vadassery (National Institute for Plant Genome Research, New Delhi, India). All microbial cultures were maintained on respective recommended media: NFb medium for AZOS, Ashby’s Mannitol Agar for AZOT, Nutrient Agar for PSB, and Potato Dextrose Agar (PDA) for PI. For the experimental purpose, a culture suspension was prepared using distilled water.

### Experimental design and plant growth conditions

The study was conducted in the greenhouse at the ICAR-DOGR, Pune, India. The soil used in this study was collected from the experimental plot of ICAR-DOGR. This site was situated at 18°32′N and 73°51′E with an elevation of 645 m above the mean sea level. The maximum air temperature ranged from 30 °C to 37 °C, whereas minimum temperature ranged from 10 °C to 17 °C with average annual rainfall of about 820 mm. The climate of the experimental site was characterized as tropical dry and humid with clay loam soil with a pH of 7.8. The ICAR-DOGR’s popular onion variety Bhima Dark Red was used in the experiment during *Kharif* 2023. Seeds were sown on raised bed during the first week of June, and seedlings were raised using the recommended fertilizer dose (RDF; 110:40:60:30 kg NPKS and 15 tons FYM per hectare) and irrigation practices to ensure healthy plant growth. New plastic pots (30 cm in height × 60 cm in length × 40 cm in width) were filled with 25 kg of garden soil, consisting of a mixture of farmyard manure and clay loam soil. At the time of transplantation, 20% of the recommended nitrogen dose, along with the full dose of phosphorus, potassium, and sulphur, was applied as a basal dose. The remaining nitrogen was applied in three splits at 15, 30, and 45 days after transplanting. Irrigation, pest, and disease management were carried out following the standard agronomic practices recommended by ICAR-DOGR in order to raise the healthy crop.

### Treatments

A pot experiment was designed using a completely randomized block design with seven treatments. Three types of control were used: blank (only soil without any plants), control (CTRL: Plants with RDF), and absolute control (ABCTRL: Plants without RDF). The treatments included: PI *+* RDF, AZOS + RDF, AZOT + RDF, PSB + RDF, Blank, CTRL, and ABCTRL. Each treatment was replicated four times. Before transplanting, 45-day-old seedlings’ roots were dipped in microbial slurry for 60 min and then transplanted in plastic pots.

### Soil sample collection

Rhizospheric soil from the depth of 15 cm and 2–2.5 mm around the onion roots were collected at the age 60 days post transplanting from each treatment in four replicates. These soil samples were processed by passing through 2.0 mm sieve before being used for further analysis. A standard protocol was used to quantify the soil pH, electrical conductivity, and concentration of available nitrogen, phosphorus, and potassium. A suspension was prepared of soil and water (1:2 ratio), and then the soil pH and electrical conductivity were recorded using a pH meter (Thermo Fisher Scientific, Waltham, MA, USA) and conductivity meter (Orion Star™ A212; Thermo Fisher Scientific, Waltham, MA, USA), respectively. The alkaline permanganate method was used to estimate the soil available nitrogen. The phosphorus concentration was calculated using Olsen’s method and the potassium concentration was calculated using the ammonium acetate method (Jackson, 1967), and the results are presented in Table 2.

### Physiological and biochemical traits

From each treatment, phenotypic traits, namely, plant height, number of leaves per plant, and leaf area were recorded at 55 days after transplanting when the plant was in the full growth stage. The fourth fully expanded leaf was used for measuring the leaf area using a portable leaf area meter and expressed as leaf area per plant. At the same time, photosynthesis (μmol CO_2_ m^−2^s^−1^), stomatal conductance (μmol m^−2^s^−1^), and leaf canopy temperature were recorded in bright sunshine hours (10.30 to 11.00 noon) using a portable IRGA (Analytical Developmental Company, Hoddeson, UK). Each observation was recorded four times from each replication per treatment. The total chlorophyll content was quantified from the leaf samples spectrophotometrically using the non-maceration method described by Hiscox & Israelstam (1979). The total chlorophyll content was quantified using the formula given by Arnon (1949):

${\rm Total\; chorophyll} = {{\left( {0.2\, *\, {\rm OD}645\,+\,8.02 \,* \,{\rm OD}668} \right)\,\times\,\left( {{\rm volume\; of\; extract}} \right)\,\times\, \left( {{\rm weight\; of\; sample}} \right)} \over {1000}}$

where OD 663 is the absorbance at 663 nm and OD 645 is the absorbance at 645 nm.

The total antioxidant activity was estimated from the leaf sample using the ferric ion reducing antioxidant power (FRAP) assays given by Benzie & Strain (1999) using a UV-visible spectrophotometer. Ascorbic acid was used as the standard. The FRAP values were expressed as mg ascorbic acid equivalents per g of sample dry weight. Total phenolic content was quantified from the bulb samples by using the Folin Ciocalteu reagent by using the method described by Singleton & Rossi (1965) with slight modification suggested by Ateeq et al. (2023). The total phenolic content was expressed as mg gallic acid equivalent (GAE) per gram dry weight of the sample. The total flavonoid content was quantified using aluminium chloride colorimetric method given by Chandra et al. (2014) and expressed as mg quercetin equivalent per gram dry weight of the sample.

### Metagenomics analysis

For the metagenomics analysis, we used seven treatments comprising PI *+* RDF, AZOS + RDF, AZOT + RDF, PSB + RDF, Blank, CTRL and ABCTRL. Rhizosphere soil was collected after 60 days of transplanting. The soil was collected in triplicate from each treatment and then combined in equal amounts. Soil samples are immediately sent for metagenomic sequencing to a commercial firm (Lifecell International Ltd, Chennai, India).

### DNA isolation, sequencing, and QC

Rhizosphere soil from the plants of each treatment was collected and DNA was extracted from the soil using a commercial kit (Macherey-Nagel, Düren, Germany) as per the manufacturers’ protocol. The concentration and purity of the extracted DNA were measured with a NanoDrop spectrophotometer (Thermo Fisher Scientific, Wilmington DE, USA). PCR amplification of the V3–V4 hyper-variable regions of the 16S rRNA gene was conducted using Phusion® High-Fidelity PCR Master Mix. The 16S rRNA sequencing was done at the Life Cell International Pvt Ltd on the Illumina Miseq platform (300PE) with 0.1 million reads per sample. The raw sequencing data quality was checked using FastQC and MultiQC software before analysis (Table 1).

**Table 1:** Summary of the raw metagenome sequencing data.

S. No.	Sample ID	No. of reads	Read length	GC %	% Q30
1.	Azospirillum	470,434	301	59	75.9
2.	Azotobacter	534,412	301	59	76.9
3.	*P. indica*	371,984	301	58	76.2
4.	PSB	348,172	301	58	76.6
5.	Blank	492,456	301	59	76.5
6.	Control	468,684	301	59	76.8
7.	Absolute control	370,324	301	58	75.7

### 16S rRNA sequence analysis

The analysis of the 16S rRNA gene amplicon sequences was conducted using the QIIME2 platform (version 2023.5; Bolyen et al., 2019). Raw FASTQ reads were quality filtered and denoised using the DADA2 plugin (version 2023.5; Callahan et al., 2016) within QIIME2 to generate high-resolution amplicon sequence variants (ASVs). The taxonomic classification of the ASVs was performed using a pre-trained naive Bayes classifier trained on the SILVA 138 reference database (release 138.1, 99% OTUs, full-length sequences; Quast et al., 2012). The resulting ASV table, containing both taxonomic identities and abundance data, was exported for downstream diversity and statistical analyses.

### Microbial community analysis

Microbial community analysis was performed using R (v4.2.3; R Core Team, 2023) and the associated packages. ASV count data were processed using the Phyloseq package (v1.42.0; McMurdie & Holmes, 2013) to assess microbial diversity and composition. Rarefaction curves were generated using the vegan package (v2.6-4; Oksanen et al., 2007) to evaluate the sequencing depth. Community composition at the phylum level was visualized through bar plots generated using microViz (v0.11.0; Barnett, Arts & Penders, 2021). Alpha diversity was calculated using the Shannon diversity index and visualized as box-plots using the Phyloseq, while beta diversity was assessed using principal coordinate analysis (PCoA) based on Bray-Curtis dissimilarity.

### Statistical analysis

The data collected for all the study parameters were subjected to a one-way analysis of variance (ANOVA). The analysis was conducted using the R statistical software (R Core Team, 2023), and the results are presented as mean ± standard deviation (SD) based on four replications. To compare treatment means, the least significant difference (LSD) test was applied at the 5% level of significant (*p* < 0.05), following ANOVA, to identify statistically significant differences among the treatments (Gomez & Gomez, 1984). To further investigate the microbial community structure, a Principal Component Analysis (PCA) and a species-level Redundancy Analysis (RDA) were performed using Hellinger-transformed abundance data with the microViz package. For RDA, the experimental variables, including Onion, PSB, and Azotobacter, were included as explanatory constraints. A non-metric multidimensional scaling (NMDS) plot was also constructed at the phylum level using Bray-Curtis distance and Hellinger transformation. Pearson correlation analysis was conducted between the top 15 taxa and microbial treatments to explore the potential associations between microbial composition and experimental conditions.

## Results

### Plant growth analysis

**Figure 1:**
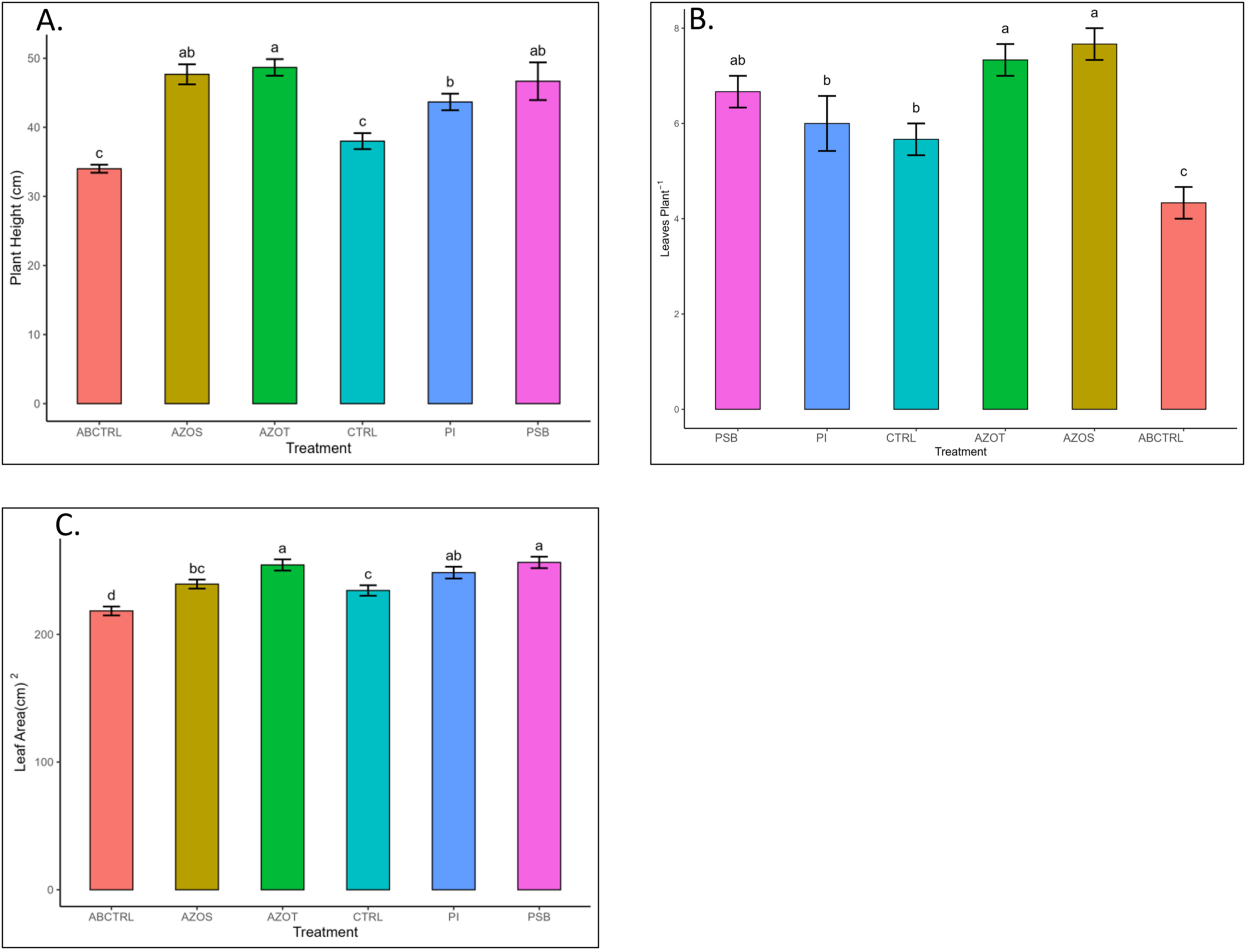
(A–C) Plant height, leaf number, and leaf area/plant in response to different microbial treatments. Pi, *Piriformospora indica*; AZOS, Azospirillum spp.; AZOT, Azotobacter spp.; PSB, Phosphorus solubilizing bacteria spp.; CTRL, control with the recommended dose of fertilizer (RDF) without microbial inoculation; ABCTRL, control without RDF and microbial inoculation. Values with different lowercase letters indicate significant differences (*p* < 0.05) as assessed using the least significant difference test.

**Figure 2:**
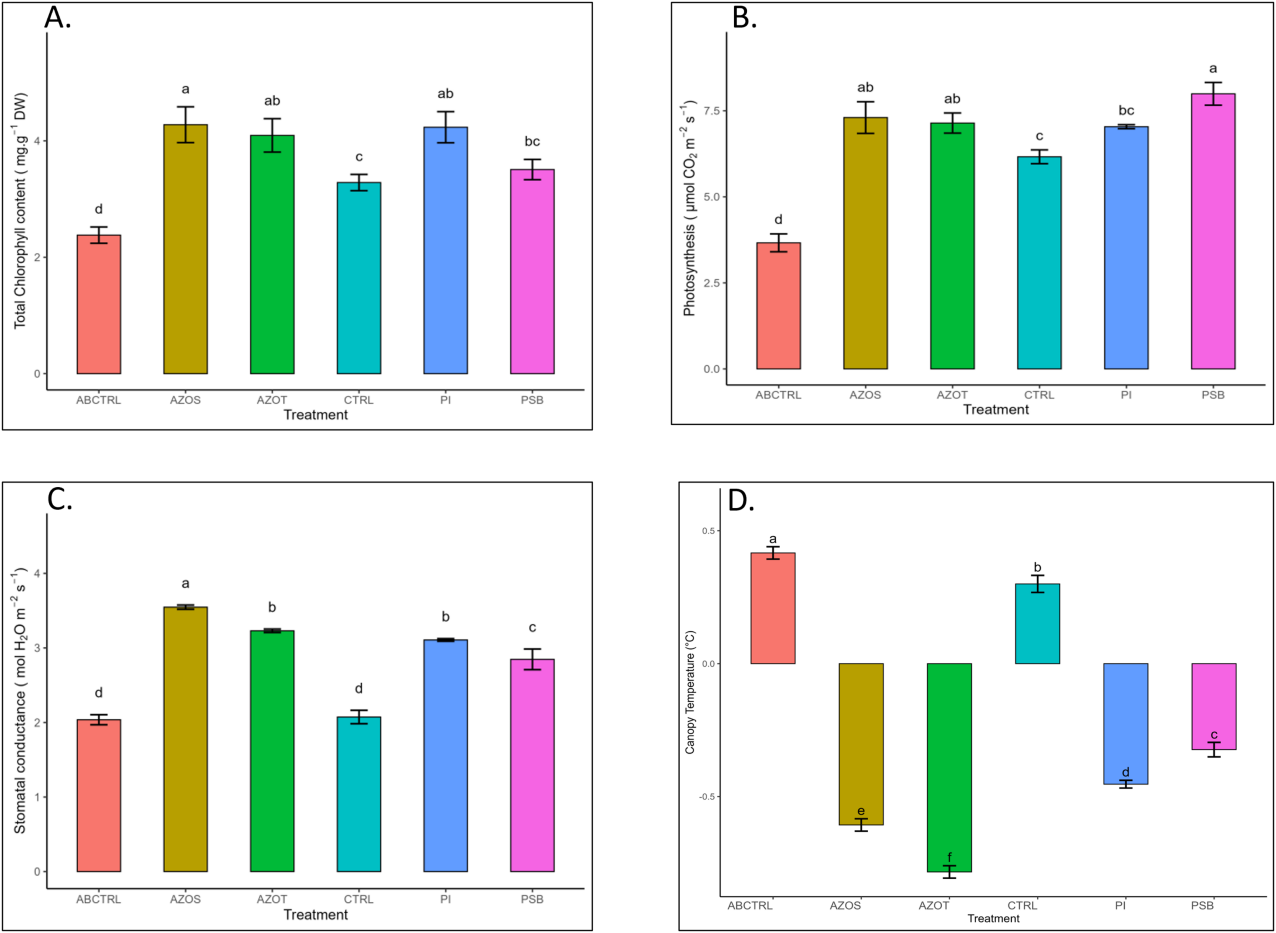
(A–D) Chlorophyll content, net photosynthesis, stomatal conductance, and canopy temperature depression in response to different microbial treatments. Pi, *Piriformospora indica*; AZOS, Azospirillum spp.; AZOT, Azotobacter spp.; PSB, Phosphorus solubilizing bacteria spp.; CTRL, control with the recommended dose of fertilizer (RDF) without microbial inoculation; ABCTRL, control without RDF and microbial inoculation. Values with different lowercase letters indicate significant differences (*p* < 0.05) as assessed using the least significant difference test.

**Figure 3:**
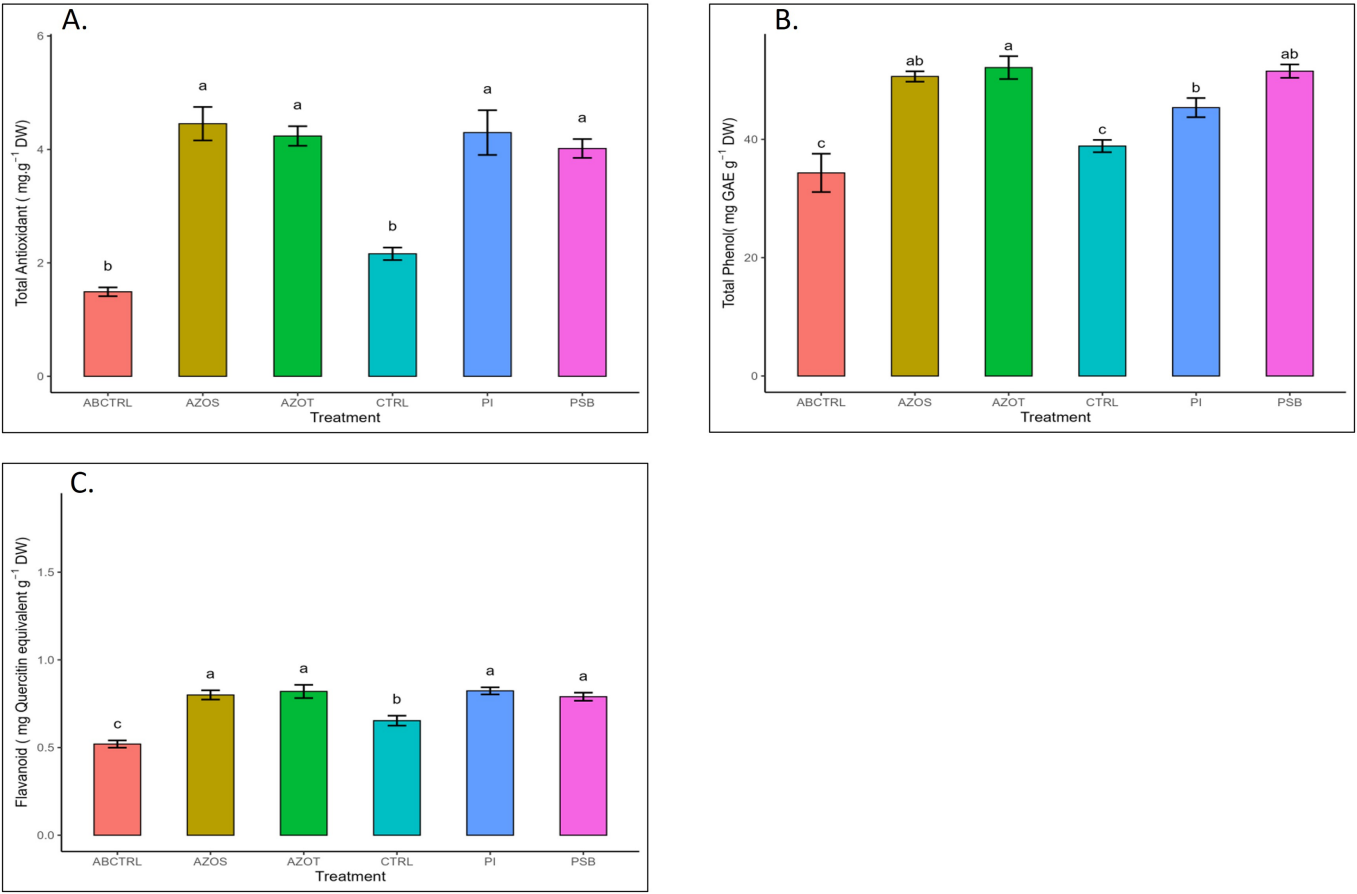
(A–C) Antioxidant enzyme activity and total phenolic and flavonoid content in response to different microbial treatments. Pi, *Piriformospora indica*; AZOS, Azospirillum spp.; AZOT, Azotobacter spp.; PSB, Phosphorus solubilizing bacteria spp.; CTRL, control with the recommended dose of fertilizer (RDF) without microbial inoculation; ABCTRL, control without RDF and microbial inoculation. Values with different lowercase letters indicate significant differences (*p* < 0.05) as assessed using the least significant difference test.

Data represented in Fig. 1 shows the response of microbial treatments to different plant phenotypic traits. Plants with microbial inoculation showed significantly higher morphological growth compared with the control (100% RDF and no microbial inoculation) and absolute control (No fertilizer and microbial inoculation). However, the growth responses exhibited by the diverse microbial inoculants were different. As depicted in Fig. 1A, microbial treatment specifically inoculation with Azotobacter spp. resulted in significantly higher plant height (48 ± 1 cm) in comparison to control (37 ± 1 cm) and absolute control (34 ± 1 cm). The minimum number of leaves per plant (5 ± 1) was recorded in untreated control plants, whereas the highest leaf number was observed in Azospirillum inoculated plants (8 ± 1) as shown in Fig. 1B. Pre-treatment of microbial inoculants significantly increased the leaf area by 9.4% to 17% compared to the control and absolute control without microbial inoculation (Fig. 1C). The significantly highest leaf area was recorded in plants inoculated with PSB (256 ± 1 sq.cm) and *Azotobacter spp* (254 ± 1 sq.cm). Overall enhancement in plant height, leaf number, and leaf area was observed in all microbial-treated plants in comparison to untreated control plants. Plants exposed to microbial treatment stimulate their chlorophyll biosynthesis process compared with the non-inoculated plants. The total chlorophyll content was found to be higher in the leaves of microbial-treated plants compared to their control relatives, as reflected in Fig. 2A. The significantly highest chlorophyll level was observed in the leaves of Azospirillum-inoculated plants (4.2 to 4.5 mg g^−1^DW) followed by Pi and Azotobacter-inoculated plants compared with the control (2.3 to 3.3 mg g^−1^DW). Along with the stimulation of photosynthetic pigment production, microbial inoculation in parallel improved the leaf gas exchange traits. Significantly more photosynthesis (49%) and stomatal conductance (55%) were recorded in the microbial inoculate plants compared to the control and absolute control (Figs. 2B, 2C). Maximum photosynthesis was recorded from the leaves of PSB-inoculated plants (7.9–8.0 μmol CO_2_ m^−2^s^−1^) whereas stomatal conductance from Azospirillum-inoculated plants (3.5 μmol m^−2^s^−1^). Minimum photosynthesis was quantified from the leaves without microbial and fertilizer treatment (3.6 μmol CO_2_ m^−2^s^−1^) as shown in Figs. 2B, 2C. Canopy temperature depression (CTD) was found to be more negative in response to microbial treatment (−0.5) compared with the control relatives (Fig. 2D). Significant variation was recorded for the CTD value in response to all the treatments. A low CTD value was observed for the leaves of Azotobacter-inoculated plants (−0.78). Biochemical traits, namely, antioxidant enzyme activity, total phenolic, and flavonoid content, improve in response to microbial treatment. Significantly more antioxidant enzyme activity was recorded in the microbial-inoculated plants (130–135% more) compared with their control untreated plants (Fig. 3A). However, a significant difference was not recorded among the microbial treatment for antioxidant enzyme activity. Maximum enzyme activity was recorded from the leaves of Azospirillum-inoculated plants (4.45 mg g^−1^DW) followed by Pi and Azotobacter. The lowest antioxidant enzyme activity was recorded from the control and absolute control plants without any microbial treatment. Enhanced levels of total phenolic and flavonoid content were recorded in bulbs harvested from microbial-treated plants. Significant variation was recorded for the bulb phenol content among microbial-treated and untreated plants. The highest phenol content was recorded in response to Azotobacter and PSB treatment (51–52 mg GAE g^−1^DW) followed by Azospirillum (Fig. 3B). A slight increment in the bulb flavonoid content was recorded in response to microbial treatment compared with their control plants. However, a non-significant variation was observed for the flavonoid level in response to different microbial treatments (Fig. 3C). The raw data for all graphs is provided in the Table S1.

### Soil analysis

Physicochemical analysis of the soil samples revealed an improvement in the soil nutrient status in response to microbial treatment. Soil pH and EC showed non-significant slight variation in response to different treatments (Table 2). Microbial inoculation increased the soil available nitrogen (137–52 mg kg^−1^) compared with their control relatives (112–136 mg kg^−1^). Maximum was recorded in the Azotobacter-inoculated soil sample (152 mg kg^−1^). Significantly high available phosphorus was recorded from the PSB-inoculated soil sample (33 mg kg^−1^) followed by *P. indica* treatment (Table 2). Variation was also recorded for the potassium level, which was found to be highest in the microbial-treated soil compared to the untreated soil, as depicted in Table 2.

**Table 2:** Physicochemical properties of the soil in response to different treatments.

Soil properties	Pre-planting	60 days after transplantation					
		AZOT	AZOS	PI	PSB	CRTL	ABCRTL
Soil pH	7.53	7.45	7.41	7.28	7.43	7.58	7.49
EC (dS m^−1^)	0.28	0.27	0.28	0.27	0.29	0.26	0.25
N (mg kg^−1^)	90.63	152.03	142.48	146.52	137.23	136.12	112.41
P (mg kg^−1^)	7.73	28.75	30.98	32.71	33.24	25.52	4.56
K (mg kg^−1^)	217.83	231.22	247.31	233.28	230.67	225.69	135.10

**Note:**

PI, *Piriformospora indica*; AZOS, Azospirillum spp.; AZOT, Azotobacter spp.; PSB, Phosphorus solubilizing bacteria spp.; CTRL, control with recommended dose of fertilizer (RDF) without microbial inoculation; ABCTRL, control without RDF and microbial inoculation; EC, electrical conductivity; N, available nitrogen; P, available phosphorus; K, available potassium.

### Rhizosphere microbiome responses to microbial inoculation

#### Impact of microbial inoculation on rhizosphere microbial richness and diversity in onion

**Figure 4:**
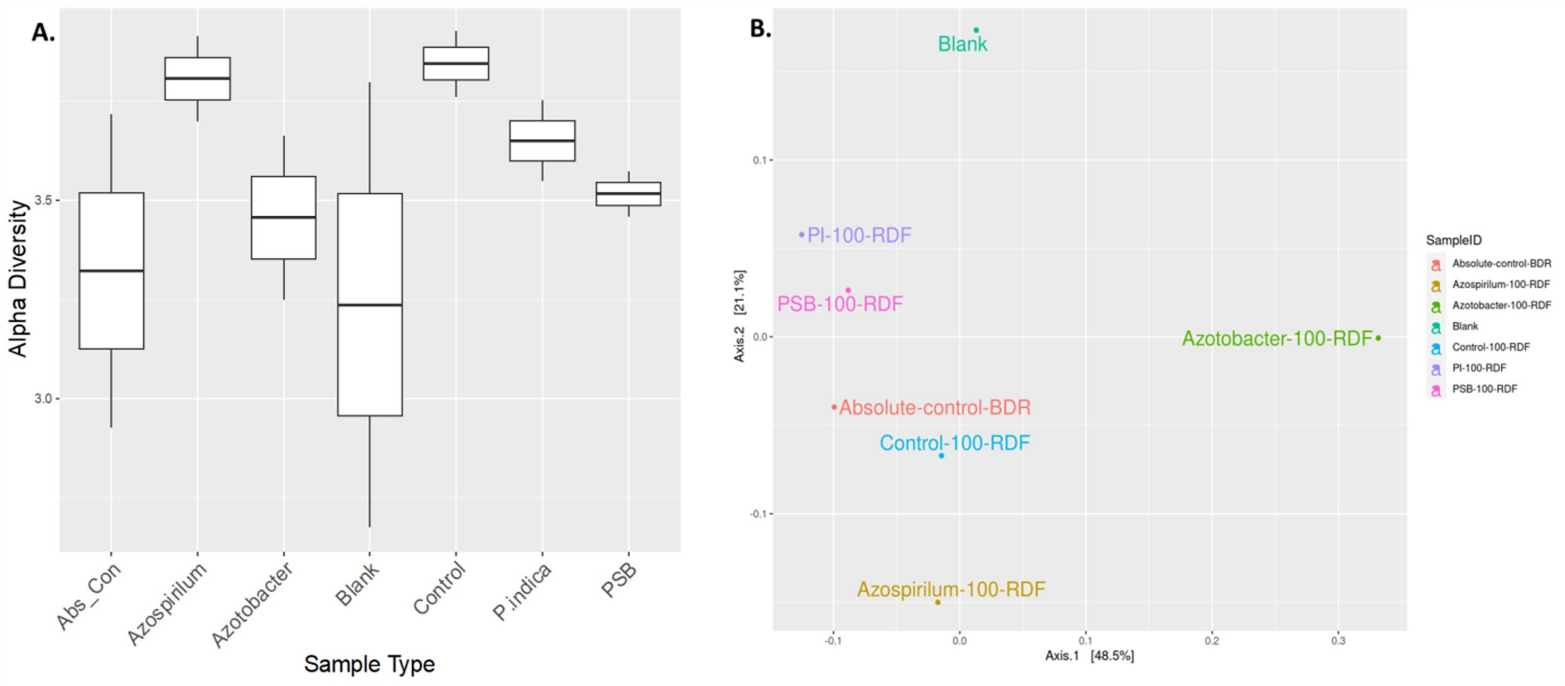
Effect of microbial treatments on soil microbial diversity in the onion rhizosphere. (A) Box plot of sample-specific alpha diversity, (B) PCoA plot with Bray-Curtis distances. PI, *Piriformospora indica*; PSB, Phosphorus solubilizing bacteria spp.; Blank, pre-planting soil without plant and microbial inoculation; Control, recommended dose of fertilizer (RDF) without microbial inoculation; Absolute control, without RDF and microbial inoculation.

The alteration in the bacterial diversity of the onion rhizosphere based on the metagenomics data was analysed using Rarefaction curve, Beta and Alpha diversity indices. Rarefaction curves were used to assess species richness (alpha diversity) and sequencing depth sufficiency in the microbiome samples. Azospirillum samples were found to have higher species richness with adequate sequencing depth. Blank soil (soil without plants and microbial inoculation) showed significantly lower diversity, which indicated the presence of fewer microbes compared to other treatments (Fig. S1). Alpha diversity is a measurement of the richness and relative abundance of bacteria within the sample. According to the results, rhizosphere bacterial alpha diversity (Chao, Ace, Shannon, Simpson, InvSimpson, Fisher) indices were significantly (*p* ≤ 0.05) affected by microbial treatment (Fig. 4A). The Azospirillum treatment exhibited the highest overall richness and moderate evenness. It consistently performed well across all metrics, suggesting that it promotes high microbial diversity, while the lowest diversity was observed in the blank and absolute control treatment (plants without RDF and microbial inoculation). This suggested the impact of microbial treatment on the soil microbial diversity.

Beta diversity analysis was performed using PCoA to visualize the similarities or dissimilarities between microbial communities based on their composition. The PCoA plot suggested that microbial treatments influenced the soil microbial community composition in response to different treatments (Fig. 4B). The first two principal coordinates explained 48.5% and 21.1% of the variation, respectively, cumulatively accounting for 69.6% of the total variance in the microbial community structure. The absolute-control and control samples were clubbed together, implying relatively less microbial diversification compared to the microbial treatments. A blank sample (presumably soil without plants and microbial inoculation) was plotted separately. Overall, the Azotobacter and Azospirillum treatments resulted in the most pronounced microbial shifts, indicating their potential role in significantly reshaping the onion rhizosphere microbiome.

#### Impact of microbial inoculants on the rhizosphere microbial community structure in onion

**Figure 5:**
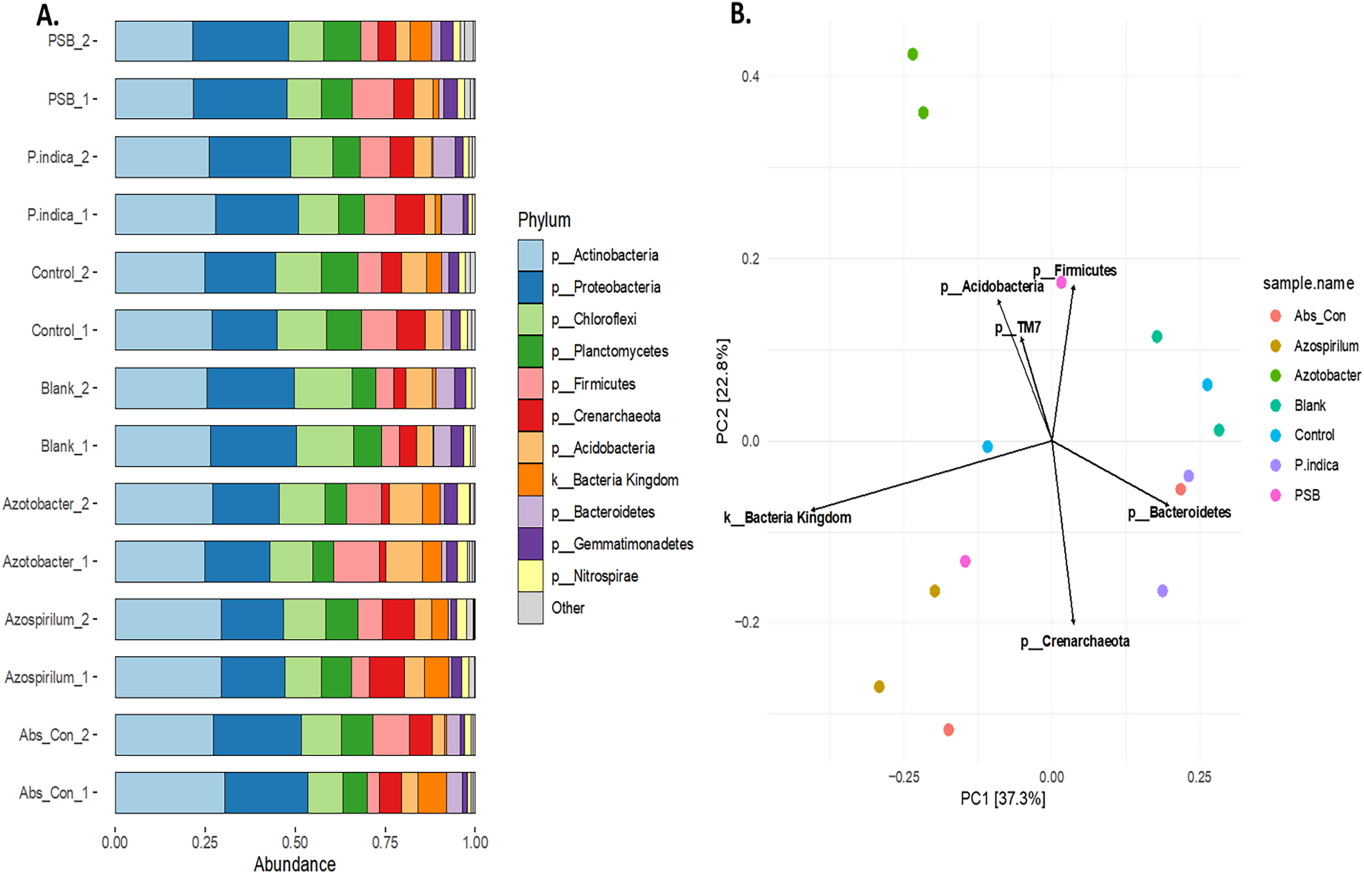
Effect of different microbial treatments on soil microbial diversity and richness in the onion rhizosphere. (A) Abundant phyla in the onion rhizosphere after microbial treatments. (B) Phylum level PCA plot with Hellinger transformation. *P. indica*, *Piriformospora indica*; PSB, Phosphorus solubilizing bacteria; Blank, pre-planting soil without plant and microbial inoculation; Control, recommended dose of fertilizer (RDF) without microbial inoculation; Absolute control, without RDF and microbial inoculation.

To study the effect of different microbes on the rhizosphere microbiota in onion, the V3 and V4 hyper-variable regions of the 16S rRNA NGS sequencing of the rhizosphere soil were performed after the microbial treatments. The composition of the rhizobium was analysed at different taxonomic levels (Table S2). First, we classified the microbiome into bacteria and Achaea; 94.5% of the reads belonged to kingdom bacteria and 5.5% belonged to Achaea. At the phylum level, a total of 17 microbial phyla were identified from the sequencing data. The top ten phyla are *Actinobacteria, Proteobacteria, Firmicutes, Chloroflexi, Planctomycetes, Acidobacteria, Crenarchaeota, Bacteroidetes Gemmatimonadetes* and *Nitrospirae* (Fig. 5A*)*, which constitute more than 98% of the total microbiota of the onion rhizosphere. Among these, four dominant phyla accounted for more than 70% of the total microbiome. *Actinobacteria* was the most abundant, representing 25.3% of the total reads, followed by *Proteobacteria* (22.2%), *Firmicutes* (12.8%), and *Chloroflexi* (11.02%). The relative abundance of these phyla varied across different treatments. *Actinobacteria* was the dominant phylum in the absolute control, Azospirillum, control, and *P. indica* treatments. In contrast, *Proteobacteria* was most abundant in the blank and PSB treatments, while *Firmicutes* was dominant in the Azotobacter treatment. The dominant phyla in the majority of treatments were consistent, but their relative abundance was found to vary across the microbial treatments. At the class level, 59 taxonomic classes of bacteria were identified in the 16S rRNA metagenomics data set. *Alphaproteobacteria*, *Bacilli*, *Actinobacteria*, *Thermoleophilia* and *Planctomycetia* are the top five dominant classes. At the genus level, a total of 177 genera were identified from the present study. The highest number of genera found in this study were from the phylum *Proteobacteria* (39) followed by *Chloroflexi* (35), *Actinobacteria* (23), *Acidobacteria* and *Bacteriodetes* (15). It was observed that the genus *Candidatus Nitrososphaera* (phylum; Crenarchaeota) was most prevalent in Azospirillum-treated soil (11.51%). The genus *Ammoniphilus* (phylum; Firmicutes) was most abundant (15%) in Azotobacter, whereas the genus *Pseudomonas* (phylum; Proteobacteria) was found to be abundant in soil treated with PSB (10.63%). At the species level, the most abundant species found to be Gargensis (Genus; *Candidatus Nitrososphaera*) belonged to the phylum *Crenarchaeota*. Phylum-level PCA plot with Hellinger transformation revealed that PC1 explains 37.3% and PC2 explains 22.8% of the total variance (Fig. 5B). To explore the microbial community composition at the different levels *viz*. class, order, family, genus, and species, a PCA plot with Hellinger transformation was generated using microViz v 0.11.0, revealing distinct clustering patterns among the samples (Figs. S2, S3).

**Figure 6:**
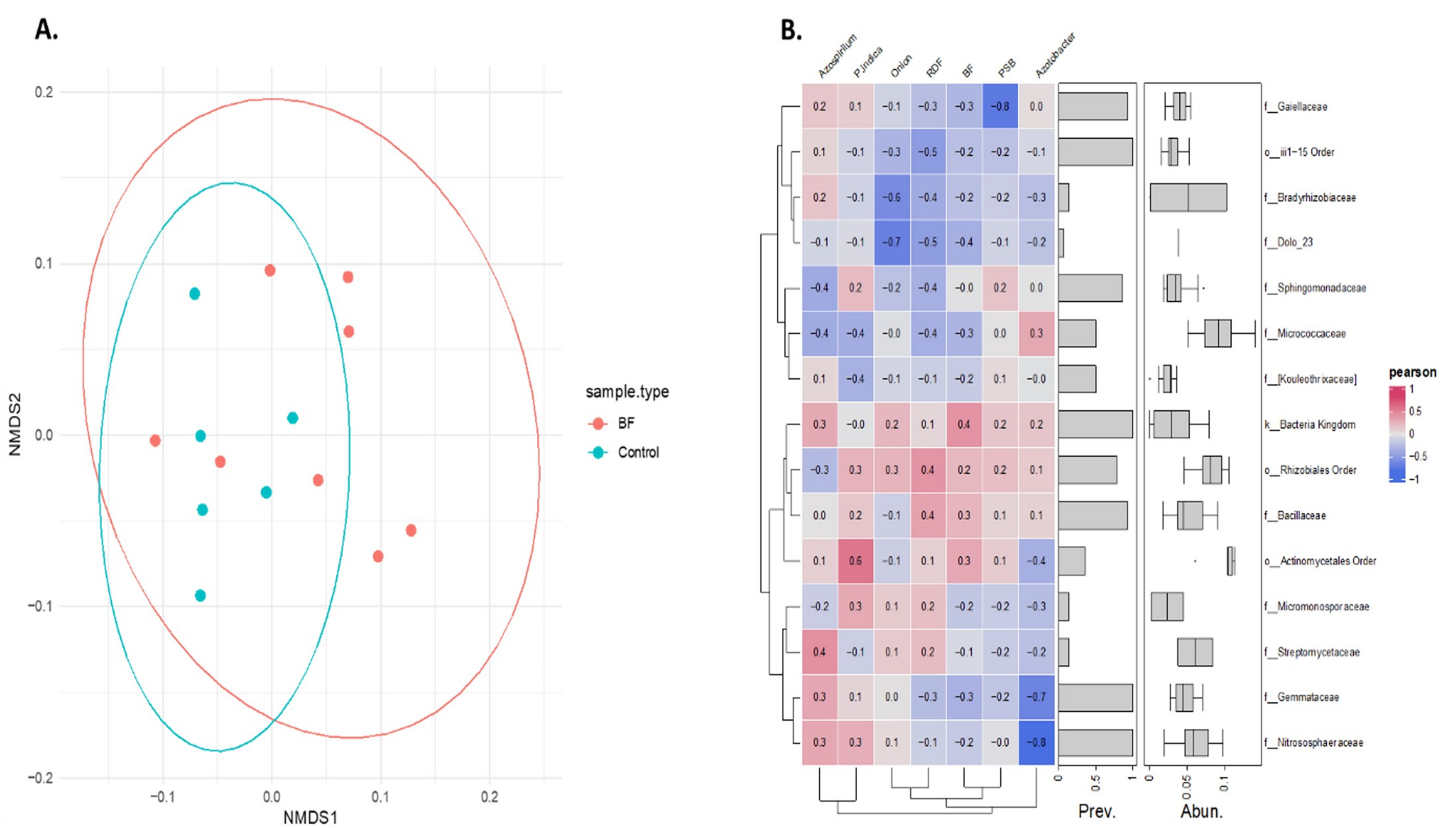
Effect of different microbial treatments on soil microbial composition and their association in the onion rhizosphere. (A) Phylum-level NMDS ordination of the onion rhizosphere microbiome based on Bray-Curtis distances and Hellinger transformation. (B) Pearson correlation heatmap of the top 15 onion rhizosphere microbial taxa and microbial treatments. *P. indica*, *Piriformospora indica*; PSB, Phosphorus solubilizing bacteria; Blank, pre-planting soil without plant and microbial inoculation; Control, recommended dose of fertilizer (RDF) without microbial inoculation; Absolute control, without RDF and microbial inoculation.

Non-metric multidimensional scaling (NMDS) analysis based on Bray-Curtis dissimilarity of Hellinger transformed phylum level data revealed a distinct microbial community pattern across treatments (Fig. 6A). Control samples (cyan) are clustered closely together, indicating higher similarity among them, while microbial-treated samples (red) are more spread out, forming a distinct group and occupying various positions along both NMDS axes. This indicates that microbial treatments resulted in a more diverse microbiome composition compared with the control samples, as indicated by their spread and distinct grouping in the NMDS analysis. Partial overlap between the red and cyan ellipses indicates some shared phyla, but overall, the microbial communities are differentiated based on the treatments.

Pearson correlation analysis revealed distinct microbial responses to different microbial treatments. PSB showed a strong negative correlation with *Gaiellaceae* (r = −0.8), suggesting the suppression of this family. Azospirillum was positively correlated with *Streptomycetaceae* (0.4), *Nitrososphaeraceae* (0.3), and *Gemmataceae* (0.3), indicating a potential stimulatory effect. In contrast, Azotobacter exhibited strong negative associations with *Gemmataceae* and *Nitrososphaeraceae*. *P. indica* showed a moderate positive correlation with the order *Actinomycetales*. These trends highlight the differential influence of each microbial treatment on the soil microbial communities.

## Discussion

Soil health, characterized by its physiochemical and biological properties, is closely linked to crop productivity. Microbial composition, abundance and diversity are the key indicators of soil health (Xing, Wang & Mustafa, 2025). Thus, soil health management is a pivotal necessity for the maintenance of soil microbial diversity and for sustainable agriculture production (Pahalvi et al., 2021). However, the continuous utilization of chemical fertilizers to meet the increasing food demand leads to soil deterioration by effecting its physical structure (Raza et al., 2020), chemical properties (Yang et al., 2018) and declining the population of beneficial soil microbial communities (Pahalvi et al., 2021). To maintain soil health, the integrated use of chemical and organic fertilizer along with the use of effective plant growth-promoting microbes (PGPM) could be an effective strategy to enhance soil health and crop yield. Likewise, to meet the challenges of onion production, there is a need to improve the nutrient management practices to boost its productivity. The aim of this study was to evaluate the effect of PGPM on the native microbial populations of the onion rhizosphere and their subsequent influence on crop growth and development. Onion seedlings were inoculated with PGPM such as, *Azospirillum brasilens* (AZOS), *Azotobacter chroococcum* (AZOT), phosphorus solubilizing bacteria (*Bacillus megaterium*) (PSB) and *Piriformospora indica* (PI) along with the recommended dose of fertilizers (RDF) before transplanting. The results demonstrated a considerable increment in plant growth in response to microbial treatments compared to untreated control and absolute control treatment. Significant variation was recorded for plant height, leaf number and leaf area with the highest being recorded in AZOT and AZOS treatments followed by PSB and PI. This enhancement in phenotypic growth may be attributed to their capacity to improve soil nutrient status, particularly by nitrogen fixation, phosphate solubilization, and production of phytohormones such as indole-3-acetic acid (IAA), cytokinins, and gibberellins that stimulate plant growth and root development (Sumbul et al., 2020; Somers, Vanderleyden & Srinivasan, 2004). AZOT, AZOS, and PSB are free-living nitrogen-fixing and phosphate solubilizing microbes that improve the composition of beneficial soil microbes. They positively alter plant growth by increasing nitrogen accumulation in plants, which improves plant phenotypic growth. In the tomato crop, Azotobacter inoculation improves plant height and biomass by increasing the N_2_ fixation and stimulating auxin, gibberellins, and phenol production (Govedarica, Milic & Gvozdenovic, 1993). Similar findings were reported in rice crop, where AZOS inoculation improved plant height, number of leaves, leaf area and biomass attributed to various adaptive mechanisms such as nitrogen fixation, phosphorus solubilization and production of phytohormones (Kaur et al., 2025). Furthermore, PSB inoculation increases the availability of fixed phosphorus from the soil for crop uptake, which improves growth and yield, as reported in sorghum crops (Sharma et al., 2013). Similarly, PI is an endophytic fungus that improves plant growth, biomass, yield, and tolerance to various biotic and abiotic stresses (Franken, 2012). In our study, all the microbial-treated plants showed increased photosynthetic activity due to high chlorophyll and stomatal conductance. According to the results, AZOT, AZOS, and PI inoculated plants maintained high chlorophyll levels, photosynthesis, and antioxidant enzyme activity compared with the control treatments. This increment is due to the high stomatal conductance and low leaf temperature that may additionally support the photosynthetic system and prevent damage due to oxidative stress. AZOT and AZOS species help in nitrogen fixation and promote nitrogen uptake and availability to plants, thereby retaining the chlorophyll level and maintaining enzyme activity with better photosynthesis efficiency. The current findings are in line with the outcomes of increased chlorophyll levels in the maize crop with AZOT inoculation (Moeinnamini et al., 2024). Similarly, the use of AZOS promotes the uptake of plant micronutrients such as zinc, iron, and manganese, which are required in very minute amounts for plant growth but are highly essential to sustain the several metabolic processes such as photosynthesis (Housh et al., 2021a, 2021b). AZOS stimulates the photosynthesis process of maize crop by promoting the uptake of manganese, an essential cofactor of oxygen evolving complex of the photosynthetic system (Housh et al., 2022). In the onion crop, AZOS inoculation showed a positive impact on growth traits such as plant height, dry matter production and chlorophyll content in relation to untreated plants (Pellegrini et al., 2021). PI increases the chlorophyll level, photosynthetic rate and stomatal conductance and improves the antioxidant defence system that promotes plant growth and development (Liu et al., 2022a). Bilal et al. (2021) reported that the inoculation of mungbean seeds with PSB recorded the maximum photosynthesis rate, number of leaves, and yield compared to untreated control plants. Biochemical analysis of harvested bulbs showed a significant increase in phenol content in response to microbial treatment, with the highest being recorded from AZOT-treated plants followed by AZOS and PSB. Our results are consistent with previous reports by Pellegrini et al. (2021) in onion crop where inoculation with AZOT improved the bulb phenol content. This positive result may be linked with increased nutrient uptake and production of auxins, which might improve the bulb quality parameters. In line with previous findings, studies indicate that the use of PGPM or biofertilizers is known to enhance the plant growth, yield and soil fertility along with enhancing crop tolerance to several biotic and abiotic stresses in different crop species (Zaidi et al., 2015; Fu et al., 2017).

Additionally, it has been established that biofertilizers change the biodiversity of the soil microbiota (Huang, 2018; Dal Cortivo et al., 2020; Liu et al., 2022b). The application of biofertilizers markedly influenced both the richness and structure of the rhizosphere microbial community. Treatments with AZOS and PSB exhibited higher alpha diversity indices, such as Shannon and Chao1, suggesting a more species-rich and evenly distributed microbiome. Increased alpha diversity is often associated with greater functional redundancy and ecological resilience, which may contribute to improved nutrient cycling and plant health. Beta diversity analysis, based on Bray-Curtis dissimilarity and visualized *via* PCoA, revealed distinct clustering of microbial communities among different treatments. This separation highlights that each biofertilizer induced a unique microbial assembly, reflecting its specific influence on the rhizosphere conditions. These results are in congruence with earlier studies that reported the alteration in alpha and beta diversity indices after treatment of biofertilizers treatments in different crops (Li et al., 2023b; Coniglio et al., 2022; Yasuda et al., 2022; Liu et al., 2020).

The dominant phyla *Actinobacteria*, *Proteobacteria*, and *Firmicutes* played distinct but complementary roles in shaping the rhizosphere microbiome under biofertilizer treatments. *Actinobacteria*, abundant in PI and AZOS treatments, are associated with organic matter decomposition and pathogen suppression. *Proteobacteria* enriched in PSB-inoculated soils likely enhanced nutrient mobilization and root colonization *via* taxa like *Pseudomonas* and *Bacillus*. *Firmicutes*, particularly in the AZOT treatment, may have contributed to the improved bulb yield through their well-known plant growth-promoting and stress-resilient traits. These are major phyla reported from the rhizosphere of several plants such as apple, maize, and rice (Munoz-Ramirez et al., 2024; Fadiji, Kanu & Babalola, 2021; Oliveira et al., 2022). These phyla were also found to be dominant in the rhizosphere of *Allium* species: *Allium fistulosum* (Zhao et al., 2020), *Allium ascalonicum* (Rosariastuti et al., 2022), and *Allium cepa* (Simarmata et al., 2023). Zhao et al. (2020) also reported that more than 95% of the total bacterial sequences were affiliated with the top ten dominant phyla in *the Allium fistulosum* rhizosphere, which are *Proteobacteria, Actinobacteria, Acidobacteria, Bacteroidetes*, *etc*. Dominance of the members of *Actinobacteria* are known to be involved in organic matter decomposition and secondary metabolite production; these results are in agreement with Simarmata et al. (2023), who reported *Actinobacteria* dominance in *A. cepa* rhizosphere treated with endophyte-based fertilizers, suggesting their enrichment under biological amendments that enhance root microbe interaction. The phylum *Nitrospirae* was more abundant in the rhizosphere soil treated with AZOT and AZOS, accounting for over 2.5% of the total microbial community, whereas its relative abundance was notably lower in the other treatments. Supplementation of nitrogen-fixing biofertilizers can lead to an increase in *Nitrospira* populations, but the relationship is indirect and is related to the nitrogen cycle where they convert nitrite to nitrate, known as nitrification. Dobrzynski et al. (2025) reported the increased relative abundance of *Nitrospira* after a few weeks of AZOT application in a wheat crop. Similarly, high nitrogen fertilizer was also found to increase the *Nitrospira* abundance in onion (Simarmata et al., 2023). Thus, it might have led to an increase in the availability of nitrate and eventually observed the increased growth response of plants (Daims et al., 2015). The biofertilizer application generally modifies the rhizosphere microbiome by virtue of community shift, nutrient availability, competition, *etc*. These three genera, *Candidatus Nitrososphaera, Ammoniphilus* and *Pseudomonas*, were found to be most abundant in the AZOS-, AZOT- and PSB-treated onion rhizosphere. In the present study, the prevalence of *Candidatus Nitrososphaera*, a key ammonia-oxidizing archaeon (AOA), was significantly higher in the AZOS-treated onion rhizosphere, suggesting that AZOS inoculation may influence archaeal nitrifier communities and enhance nitrogen cycling. AZOS primarily promotes plant growth through nitrogen fixation and other mechanisms (Renoud et al., 2022). Although direct studies on the effect of AZOS on AOA are limited, evidence from other crops supports this interaction. For instance, maize inoculation with AZOS not only improved root growth and nitrogen uptake but also stimulated changes in nitrogen cycling microbial communities (Florio et al., 2017). AOA, including *Candidatus Nitrososphaera*, are more efficient at oxidizing ammonia under oligotrophic or biologically enriched conditions (Zhalnina et al., 2012). Their enrichment in AZOS-treated soils likely reflects a more favourable environment for archaeal nitrification. Similarly, *Ammoniphilus* was abundantly detected in the AZOT treatment, suggesting their potential role in enhancing ammonia turnover under biologically active conditions. Its presence reflects improved ammonium availability and microbial adaptation to nitrogen-rich microsites. AZOT fixes atmospheric nitrogen by converting it into ammonia, a plant-available form that can be readily absorbed and utilized for growth and development (Prajapati, Yami & Singh, 2008). A significant enrichment of *Ammoniphilus* and other beneficial flora was observed in Pakchoi by Wang et al. (2022). The genus *Pseudomonas* was enriched in PSB-treated onion rhizosphere soil, consistent with its known role in phosphate solubilization and root colonization. Consistent with findings in *Allium hookeri* (Kshetri et al., 2024), where PSB consortia bio-augmentation led to *Proteobacteria* dominance and enrichment of the genus *Pseudomonas*. *Pseudomonas* plays diverse roles in the rhizosphere, such as phosphorus solubilization and plant growth promotion, and acts as a biocontrol agent (Sah, Krishnani & Singh, 2021; Krishnashis et al., 2020).

NMDS is an unconstrained ordination method that visualizes similarities or differences in the microbial community composition between samples. In the present study, the NMDS plot based on Bray-Curtis dissimilarity revealed clear compositional shifts in the rhizosphere microbiome at the phylum level across treatments. This suggests that the application of biofertilizers contributes to the stabilization and enrichment of specific microbial phyla in the onion rhizosphere. Earlier studies have used NMDS to visualize the rhizosphere microbiome shift in response to different types of fertilizers such as biofertilizers and inorganic and organic fertilizers (Yang et al., 2019; Liu et al., 2022b; Carrascosa et al., 2023; ur Rahman et al., 2023).

The present study demonstrated that different microbial treatments distinctly influence the composition of the soil microbial communities. The strong negative correlation of PSB with *Gaiellaceae* and the contrasting effects of AZOS and AZOT on *Gemmataceae* and *Nitrososphaeraceae* highlight how specific biofertilizers can either suppress or promote key microbial taxa. The positive association of PI with *Actinomycetales*, a group that is often linked to plant growth promotion, suggests potential functional benefits. Such interactions suggest that biofertilizers not only supply nutrients but also influence microbial network dynamics (Timmusk et al., 2017). These correlations point to selective shifts in microbial structure, likely driven by nutrient availability, microbial competition, or signalling interactions induced by the biofertilizers (Mitter et al., 2019; Trivedi et al., 2020). However, further research is necessary to elucidate the underlying mechanisms and to optimize the application of biofertilizers for onion crop improvement.

## Conclusion

This research represents a novel integrative approach which links metagenomic analysis with agronomic traits to assess the impact of plant growth promoting microbes on the onion rhizosphere. The results clearly show that different plant growth microbial treatment not only improved plant growth but also brought about significant changes in the microbial community composition. Treatments such as AZOS and PSB enriched beneficial microbial groups such as *Pseudomonas*, *Streptomycetaceae*, and *Nitrososphaeraceae*. The diversity indices supported the observation of increased microbial richness in the treated soils. The correlation analysis further revealed specific microbial responses to each treatment. Overall, the study highlights how plant growth promoting microbes can be strategically used to improve both soil health and onion productivity.

While study establish a strong foundation for PGPM utilization, these findings are limited by the controlled experimental setting, which may not capture the full environment influenced variability. A crucial next step requires field validation across varied agro-climatic zones to confirm the consistency of results. Future research should employ meta-transcriptomics to elucidate active metabolic pathways and assess the long-term stability of the enriched microbial taxa within commercial fields.

## Supplemental Information

10.7717/peerj.20566/supp-1 Supplemental Information 1 Data of phenotypic observations.

10.7717/peerj.20566/supp-2 Supplemental Information 2 The composition of the rhizobium was analysed at different taxonomic levels.

10.7717/peerj.20566/supp-3 Supplemental Information 3 Apha diversity analysis.

10.7717/peerj.20566/supp-4 Supplemental Information 4 Family level PCA plot with Hellinger transformation.

10.7717/peerj.20566/supp-5 Supplemental Information 5 Genus level PCA plot with Hellinger transformation.

## Additional Information and Declarations

### Data Availability

The following information was supplied regarding data availability:

The raw sequencing data is available at NCBI SRA: PRJNA1327582.

The raw data are available in the Supplemental Files.
