# Supplementary material for: Microbial allies: shaping growth, physiology, and rhizosphere dynamics of onion (Allium cepa L.)
Source: PeerJ. 2026 Jan 6;14:e20566. doi: 10.7717/peerj.20566 (PMC12786136; doi:10.7717/peerj.20566)
Supplement: Supplemental Information 5 [file peerj-14-20566-s005.docx]

Supplementary figure 3: Genus level PCA plot with Hellinger transformation
